# Integrating a diabetes and hypertension case management package within primary health care: a mixed methods feasibility study in Bangladesh

**DOI:** 10.1186/s12913-018-3601-0

**Published:** 2018-10-23

**Authors:** R Huque, S Nasreen, F Ahmed, J P Hicks, J Walley, J N Newell, H Elsey

**Affiliations:** 1 Advancement through Research and Knowledge (ARK) Foundation, House No. B 130, Road No. 21, New DOHS, Mohakhali, Dhaka, 1206 Bangladesh; 2 Nuffield Centre for International Health and Development, Leeds Institute of Health Sciences, University of Leeds, Worsley Building, Leeds, LS2 9JT UK

**Keywords:** Non-communicable disease, Diabetes, Hypertension, Primary health care, Bangladesh

## Abstract

**Background:**

Almost three quarters of non-communicable disease (NCD) deaths, and 82% of premature NCD deaths, occur in low- and middle-income countries. Bangladesh has an estimated 7 million hypertensives and 10 million diabetics, and primary care is struggling to respond. Our aim was to develop and support implementation of a diabetes and hypertension case management package, and assess its appropriateness, feasibility and acceptability in two NCD clinics within two primary-care centres in Bangladesh.

**Methods:**

We used a convergent mixed methods design. We first assessed the level of appropriate hypertension and cardiovascular disease patient management, based on a composite outcome indicator using data from patients’ treatment cards. Appropriate management was primarily informed by International Diabetes Federation (IDF) and World Health Organisation (WHO) guidelines. We then performed qualitative in-depth interviews with doctors and patients to explain these quantitative findings and to understand the challenges to achieving appropriate patient management in the NCD clinics.

**Results:**

Eighty-one newly diagnosed patients were recruited. Over 3 months, 53.1% (95% CI 42.3% to 63.6%) of patients were appropriately managed. We found incomplete diagnosis (especially missing hypertension diagnosis alongside diabetes) and non-provision of follow-up appointments were the main causes of the relatively low level of appropriate management. We conducted interviews with 11 patients and 8 health professionals and found a shortage of human resources, reporting materials, available drugs and diagnostic equipment. This undermined patients’ willingness to attend clinics and doctors’ willingness to offer follow-ups. Hands-on skill-building training was valuable in increasing doctors’ competence for appropriate management, but was seen as a novel training method and faced constraints to implementation.

**Conclusions:**

A clinical guide, skill-based training and recording package can be implemented in routine primary care and can lead to appropriate management of around half of diabetic and hypertensive patients in a low-income country. However, considerable health systems challenges must be addressed before more patients can be managed appropriately.

## Background

Non-communicable disease (NCD) is the largest cause of mortality in the majority of low- and middle-income countries (LMICs) causing 68% of all deaths worldwide [1]. Almost three quarters of NCD deaths, and 82% of premature NCD deaths, occur in LMICs [1]. This presents a significant challenge to health services in LMICs, where the focus has traditionally been on treating acute conditions [2]. Refocusing towards chronic disease requires LMICs to make major health systems changes, including to care services at primary and community levels and prevention services [3]. Such services must include ways of following up patients on a regular basis, and referral systems allowing upward-referral and, more problematically, referral back, to ensure appropriate tertiary and community care. Health worker skills need to be re-oriented to support ongoing relationships with patients, with a focus on interpersonal skills to encourage lifestyle behaviour change [4], currently rarely available in low-resource settings [5]. This will involve substantial investment [6].

In Bangladesh the burden of NCDs now surpasses infectious diseases, and accounts for 61% of all adult deaths [7]. Approximately 12 million people, 32% of women and 19% of men, aged 35 years or older have hypertension [8]; while overall 7 million people, 7.4% of individuals aged > 15, have diabetes [9]. Very few patients with hypertension and diabetes are treated according to internationally recommended guidelines, and mostly only in specialist clinics such as Diabetic Association of Bangladesh (BADAS) hospitals/health centres, and other privately-managed specialist clinics that are too expensive for most, especially the poor [10].

Although Bangladesh has adopted national strategic plans for the prevention and treatment of NCDs [11, 12], progress has been limited [13]. Health workers in the primary health-care system are not yet trained in NCD treatment [14], the number of trained personnel in secondary and tertiary care services is inadequate, biochemical investigations required for accurate diagnosis are currently only available on a fee-for-service basis and the provision of basic drugs for treatment is limited and sporadic [10]. In addition, despite having a national essential drugs policy that includes a list of essential drugs for use in public health services, drugs for treating diabetes and Cardiovascular Diseases (CVDs) are not on the list [14]. A lack of clear institutional responsibilities, an absence of dedicated financing and competing priorities have been identified as barriers to implementing strategic plans for NCDs [14].

In response, the Government has taken several policy decisions including establishing a separate operational plan for NCDs [10], and establishing an NCD ‘corner’ (an NCD clinic room within the Upazila Health Complex), running in parallel with existing services at selected Upazila Health Complexes (UHCs) [1]. UHCs are sub-district level primary care facilities with 31 or 50 beds, posts for nine doctors, ten nurses and 23 other staff, and having 270,000 catchment population. An UHC usually has in-patient department, an out-patient department and a family planning unit which together provide basic promotive, preventive, and curative services including comprehensive emergency obstetric care (EOC) services, gynaecology, anaesthesia, nursing and basic laboratory facilities. Despite the initiative of establishing NCD corners at UHCs, the majority of NCD corners are not fully functional due to non-availability of drugs, dedicated and appropriately trained staff and adequate systems for maintaining long-term individual patient records.

To support enactment of the NCD strategy, we initiated a project to develop and support implementation of a diabetes and hypertension case management package, in close collaboration with the Government of Bangladesh. We developed an evidence-based package [15] for use by UHC doctors to diagnose and treat hypertension, type 2 diabetes, chronic obstructive pulmonary disease (COPD) and bronchial asthma, ischemic heart disease, and eye problems; and to diagnose, but not treat, cancers. A key principle within our study was to ensure the intervention was embedded within the government health system, and was therefore scalable. This meant that no additional drugs or equipment, beyond that already available within the government health system were provided.

The objectives of the study reported here were to test the package’s appropriateness, feasibility and acceptability regarding hypertension and diabetes patient management in two NCD clinics within two primary-care level UHCs. We also assessed adherence to clinical protocol for management of diabetes and hypertension.

## Methods

### Study design

We used a convergent mixed methods design [16]. We first assessed the quality of hypertension and diabetes patient management using a quantitative composite outcome indicator. We then carried out qualitative in-depth interviews (IDIs) with doctors and patients to understand our quantitative findings, and to understand challenges to achieving appropriate patient management in the NCD clinics.

### Setting

We purposively selected UHC NCD clinics for this feasibility study. The government-prescribed essential service package (ESP) at UHC level includes maternal health, nutrition, family planning and treatment of common diseases. UHCs are typically staffed with nine doctors including four specialists (gynecologist, surgery, anesthetist, pediatrics); nurses; medical assistants; sanitary inspectors; health inspectors; family planning inspectors; health assistants; technologists; and family welfare visitors/midwives.

Due to the small number of UHCs with functioning NCD clinics with an assigned doctor, necessary equipment and drugs, we were only able to select two UHCs, one in Dhaka division and one in Chittagong division. UHC1 (Dhaka division) had 7 doctors and 11 nurses and saw 150–200 patients per day; 3 private clinics operated in close proximity to the UHC. UHC2 (Chittagong) had 7 doctors and 9 nurses, and saw 280–300 patients per day; two specialist diabetes clinics provided by BIRDEM (Bangladesh Institute of Research and Rehabilitation of Diabetes, Endocrine and Metabolic Disorders), and around 30 private clinics, operated in close proximity.

### Study participants

For our quantitative study, eligible adults were aged 18 and over, newly diagnosed with diabetes and/or hypertension and not on drug treatment at the point of enrolment. We also excluded anyone found at diagnosis to have sufficiently complicated or severe disease that they required referral to the district specialist.

For our qualitative study we included patients with diabetes and/or hypertension and doctors from the NCD clinic and outpatient departments in the two UHCs. NCD doctors identified patients with diabetes and/or hypertension, who we interviewed after their routine consultation. While the sample was dependent on which patients were available on the day, the researchers aimed to interview both men and women, young and old.

### Intervention

CVD and diabetes management was primarily informed by International Diabetes Federation (IDF) [17] and World Health Organisation (WHO) guidelines [18]. A national technical working group of medical specialists in diabetes, CVD, oncology and respiratory health advised on the content of the package, ensuring its relevance to the Bangladesh health system. The package included a case management desk guide for NCD doctors; guidelines for nurses or paramedics to provide life style education on diet, exercise and tobacco cessation; a lifestyle education leaflet for patients; an individual patient treatment record card for clinicians in the NCD clinic to record ongoing treatment; a booklet for patients to record follow-up dates, drugs prescribed and instructions how to take drugs; and a training guide to be used during nationwide training by clinical specialists to all NCD doctors and nurses. All materials can be found here on our Communicable Disease-Health Service Delivery (COMDIS-HSD) website [19].

We were able to integrate training on our NCD package within the existing government training programme for UHC staff, intended to set up the NCD clinics across the country. The NCD specialist doctor and three out-patient doctors from each UHC attended the training. Our research team supported the delivery of the first batch of training provided to four UHCs, including one of our study UHCs (UHC1). The training was conducted from 21 to 23 April 2015. At our suggestion, nurses were initially included in the training, but due to doctors’ concerns over the level of prior knowledge required, it was decided that future training would only be provided to UHC doctors. The training comprised 12 sessions, four of which covered diabetes and hypertension: details are available from the authors. In brief, the intervention followed the following structure. Out-patient doctors who saw patients with suspected symptoms or clear risk factors for hypertension or type 2 diabetes referred them to the NCD corner doctor. The NCD doctor diagnosed patients according to the appropriate management guidelines in the desk guide. The patient was then asked to return for follow-up after 15 days. The NCD doctor completed the individual patient NCD card to record treatment and follow up dates. During the 3-month study period, patients were advised to return for three follow up visits.

### Outcomes

We assessed the quality of hypertension and diabetes patient management using a binary indicator of whether a patient was appropriately managed over a 3-month period following diagnosis with type 2 diabetes and/or hypertension. We classified a patient as appropriately managed (as defined in the desk guide) if:

1. they were diagnosed with type 2 diabetes and/or hypertension according to the procedure specified in the desk guide: for diabetes, random blood glucose (RBG) and fasting blood glucose (FBG) tests performed and RBG ≥11.1 mmol/L and FBG ≥7 mmol/L, and blood pressure (BP) checked twice; and for hypertension, (BP) checked twice and systolic BP > 140 mmHg and/or diastolic BP > 90 mmHg, and FBG taken or RBG (and if high then FBG taken); and
2. treatment was initiated with the appropriate drugs, if required (and variations recorded and justified); and
3. if at diagnosis the patient was found to have sufficiently complicated or severe disease that they required referral, as defined in the NCD desk guide, they were referred to the district specialist; or if referral was not required then referral to the district specialist did not occur; and
4. education and counselling was provided on the disease, treatment, adherence, and lifestyle behaviour change (as indicated by a record of attending the NCD nurse for counselling); and
5. they were given a follow-up appointment.

A deviation from the initial planned definition of appropriate management occurred, related to checking whether BP had been measured twice. While the desk guide stated two BP measurements must be taken, the patient treatment card did not specify the need, or provide a place to record the second result. As a result, none of the clinicians recorded two BP results on the patient card. Given this limitation, we adapted our outcome measure to specify appropriate diagnosis as requiring only one recorded BP.

We also created six additional outcomes to explore specific components of patient treatment quality. Five outcomes were the separate (binary) indicators that together formed the appropriate management outcome, as defined above: 1) appropriate diagnosis, 2) appropriate drug initiation, 3) appropriate referral, 4) provision of education and counselling, and 5) provision of a follow-up appointment. The final outcome was a binary indicator of whether a patient was provided with a drug that was not on the Essential Medicines List.

### Data collection procedures

All data was collected between 10th August and 31st December 2015 by researchers at the ARK Foundation. All quantitative data on the management of diabetes and hypertension were collected from the patient record card developed by the research team. One fieldworker was appointed in each UHC to collect data from the treatment card. Fieldworkers received training on data collection tools and procedures from the research team. IDIs were conducted by researchers from ARK, one with experience in qualitative research and two more junior researchers. Interviews were audio-recorded, translated into English and transcribed within two days by the interviewers.

### Analysis

We summarised patient characteristics. Outcomes were summarised as percentages with 95% confidence intervals calculated using the Wilson score interval method [20]. All analyses were performed using R (version 3.2.2). These quantitative analyses were used to inform development of the qualitative analysis.

Interviews were translated, transcribed verbatim and analysed thematically using the Framework approach [21]. IDI transcripts were coded to identify facilitators and barriers to implementation and acceptability of the intervention to patients. All of the transcripts were first checked for accuracy by the researcher who conducted the interview. Transcripts were coded by two ARK researchers and a sample of transcripts were independently coded by a third experienced qualitative researcher. We adopted the following stages of Framework analysis: familiarisation, constructing a thematic framework, indexing and charting, mapping and interpretation. NVivo 11 and Excel 2010 software packages were used for qualitative data analysis.

### Ethics

Ethical approval was received by both Bangladesh and the United Kingdom (UK) ethics committees. Written informed consent was obtained from all participants at recruitment. The information sheet was read out to all participants and those who were illiterate provided a thumb print to indicate consent.

### Sample size

We estimated that to obtain a 95% Confidence Interval (CI) at most ±10% for outcome of appropriate management, assuming at least 45% of patients were categorized as appropriately managed, required a sample size of 95. To adjust for a cluster size of 20, and an assumed intra-cluster correlation coefficient of 0.05, we estimated a sample size of 190 was needed.

## Results

### Recruitment to quantitative study

The limited availability of UHCs with operational NCD clinics meant that we were unable to recruit our planned sample of 190 patients, instead recruiting only 81 patients, which reduced the precision with which we could estimate our outcome measures. Table 1 gives baseline information on these patients.

**Table 1:** Patient characteristics at baseline by sex

	Male	Female
n	42 (51.9%)	39 (48.1%)
Age	50.33 (11.5)	43.00 (10.4)
Occupation		
Employed	12 (28.6%)	4 (10.3%)
Self-employed	25 (59.5%)	0 (0%)
Unemployed	5 (11.9%)	35 (89.7%)
Diagnosis		
Diabetes	25 (59.5%)	24 (61.5%)
Hypertension (HTN)	10 (23.8%)	12 (30.8%)
Diabetes & HTN	7 (16.7%)	3 (7.7%)
Body Mass Index (kg/m^2^)	24.09 (3.2)	24.23 (3.9)
Waist circumference (cm)	86.77 (12.8)	89.46 (18)
Systolic blood pressure (mm Hg)	135.29 (22.9)	135.64 (23.3)
Diastolic blood pressure (mm Hg)	86.55 (13)	87.44 (14.9)
Random blood glucose (mmol/L)	15.09 (5.7)	15.84 (5.8)
Fasting blood glucose (mmol/L)	9.78 (3)	10.61 (3.1)
Tobacco user		
No	22 (52.4%)	23 (59%)
Yes	20 (47.6%)	16 (41%)
Smoke tobacco	11 (26.2%)	0 (0%)
Chew tobacco	8 (19.0%)	16 (41.0%)
Both	1 (2.4%)	0 (0%)

Data are n (%) or mean (SD)

The majority of patients were diagnosed as being diabetic, with approximately half as many diagnosed as hypertensive, and a minority diagnosed as both diabetic and hypertensive. Men and women were broadly equally represented, and most patients were middle-aged, with large proportions being employed (including self-employed) or unemployed. Most patients had BMI scores at the lower end of the internationally recognised risk range [22], with no clear differences between diagnosis groups, while many used tobacco, with proportionally more patients diagnosed with hypertension using tobacco than those diagnosed with diabetes or diabetes and hypertension. Unsurprisingly those diagnosed as hypertensive or hypertensive and diabetic had the highest blood pressure values compared to those diagnosed a diabetic, and those diagnosed as diabetic or diabetic and hypertensive had the highest blood glucose values compared to those diagnosed as hypertensive.

IDIs were held with eleven patients and eight doctors, whose characteristics are given in Tables 2 and 3 respectively.

**Table 2:** Characteristics of patients interviewed

Patient Exit Client (EC) Identity (ID)	Age group (years)	Sex	Education	Occupation
UHC1 EC1	35–44	Male	Primary school (Grade 5)	Farmer
UHC1 EC2	35–44	Male	Secondary School Certificate	Office worker
UHC1 EC3	45–54	Male	No schooling	Farmer
UHC1 EC4	25–34	Female	Junior school (Grade 8)	Housewife
UHC1 EC5	35–44	Male	Primary school (Grade 5)	Fish farmer
UHC1 EC6	35–44	Male	Higher Secondary School Certificate	Office worker
UHC2 EC1	35–44	Female	Primary school (Grade 5)	Housewife
UHC2 EC2	65+ years	Female	Primary school (Grade 5)	Unemployed
UHC2 EC3	45–54	Male	Higher Secondary School Certificate	Office worker
UHC2 EC4	25–34	Female	No schooling	Housewife
UHC2 EC5	35–44	Male	Primary school (Grade 5)	Driver

**Table 3:** Characteristics of health professionals interviewed

Doctor ID	Sex	Type of training: interactive NCD training (1st batch), standard training or no training
UHC1 Upazila Health & Family Planning Officer (UH & FPO)	Male	NCD training in 1st batch
UHC1 Outpatient Department (OPD) doctor 1	Female	No NCD training
UHC1 OPD doctor 2	Male	No NCD training
UHC1 NCD doctor	Male	NCD training in 1st batch
UHC2 UH & FPO	Male	NCD training in 2nd batch
UHC2 OPD doctor 1	Male	No NCD training
UHC2 OPD doctor 2	Male	No NCD training
UHC2 NCD doctor	Male	NCD training in 2nd batch

### Experiences of implementing the NCD intervention

#### Staff training

Challenges were experienced with the provision of training to the NCD doctors and nurses. The training had a strong interactive component, with opportunities to practice diagnosing and managing diabetic and hypertensive patients, contrasting with the more didactic style normally used. Only the first batch of doctors received this full interactive training, subsequent batches receiving more traditional training (Table 3). Doctors clearly appreciated the interactive sessions. While the majority of respondents emphasised they already had a good knowledge of diagnosis and management, some thought more training would have been helpful, particularly to practice key clinical skills. In addition, most doctors said that although BP should be measured twice they only recorded the last reading; however, some admitted that when they had a heavy workload they only took BP once.

#### Staff availability

Shortages of nurses in the NCD clinics would have constrained our ability to test the intervention materials developed. Consequently, our researchers acted as nurses, supporting the NCD doctor by doing some blood glucose tests. In addition, we used our own staff to provide health education; this explains why all the 81 patients recruited received nurse (educator) referral for lifestyle advice.

Even when nurses were available, they were often taken up with other duties within the UHC. In response and in addition to the support from our staff, the NCD doctors provided some brief lifestyle advice themselves:

‘… there is no nurse here. One nurse was sent to receive the training; but could not work properly here due to the duties with in-patients. Actually, here, a doctor, a nurse and a helping person is required, but we don’t have these staff. I am doing this [managing the NCD clinic] directly. I also provide lifestyle advice.’ NCD doctor, UHC1

Doctors were often required to work in other departments in the UHC, including the outpatient department and emergency:

‘The doctor who works with us at the NCD clinic of the Upazila Health Complex stays there regularly, but he needs to work in other departments too … as there aren’t many doctors available at my health complex, we cannot allot someone specifically …’ UH & FPO, UHC1

Although the OPD doctors stated that while the doctor was away from the NCD clinic, another UHC doctor would provide cover. These doctors were unlikely to have received the specific NCD training. Despite this issue being evident in both UHCs, none of the patients questioned the doctors’ skills and only one patient from UHC2 complained the NCD doctor was not available.

#### Drug availability

The health system struggled to provide the drugs and staff needed to give adequate NCD care:

‘Actually, NCD drugs are not available. Gliclazide and metformin finishes within 2 to 4 days – but the whole month remains to cover other patients.’ NCD Doctor, UHC2

Similarly, the majority of patients could not obtain the drugs prescribed from the UHC:

‘No, they didn’t provide me the medicine. I bought it from medicine shop.’ Patient EC 3, UHC2

We therefore provided financial support to the patients at UHC1 to purchase drugs from nearby pharmacies. Initially UHC2 had adequate supplies of drugs, but we later discovered drugs ran out partway through the study.

The limited availability of drugs not only affected patients’ expenditure, but also partially explained the low number of patients attending the NCD clinic, patients attending only when they were sure drugs would be available:

‘Usually, when medicine arrives after 2 or 3 days it finishes – here patients know when the medicine has arrived.’ NCD Doctor, UHC2

#### Individual patient records

The NCD doctors all valued the treatment card. This was supposed to be distributed by the government, but supplies ran out part way through the study. When cards were available they were completed fully by doctors who appreciated this system for keeping long-term individual records:

‘I have seen such a treatment card during our training but I did not see it here, as I have started working here after the completion of your project. I think this type of treatment card should be provided by the authority for following the NCD patient properly.’ NCD Doctor, UHC2

#### Referral

Referral to specialist care did not always occur. Doctors in both UHCs said they were concerned that for the poorest patients, the costs of accessing specialist care were prohibitive. They therefore attempted to manage patients within the UHC:

‘The main barrier is the poor financial condition of the patients. When we feel that the patient is needed to be referred, we want to refer them but most of the patients are unwilling to go for the expenses.’ OPD Doctor, UHC2

Limited communication and collaboration between the UHCs and nearby specialist clinics may also have undermined referral processes:

‘I heard from the patients that there is a place … but it is very far. So, it is not possible to refer patients there and also we do not have affiliation or collaboration with them.’ NCD Doctor, UHC1

### Treatment quality

Only around half of study patients (43/81, 53.1%) were appropriately managed: incomplete diagnostic processes (especially with doctors not taking the BP of patients diagnosed with diabetes, or when they did record a raised BP, not adding the additional diagnosis of hypertension) being the primary cause, followed by doctors not providing a follow-up appointment, then incorrect drug initiation and inappropriate referral being the other, increasingly less frequent, causes (Table 4).

**Table 4:** Treatment quality indicators for NCD patient management

Indicator	n/N	% (95% CI)
Appropriate management	43/81	53.1 (42.3, 63.6)
Appropriate diagnostic process^a^	57/81	70.4 (59.7, 79.2)
Appropriate drug initiation	74/81	91.4 (83, 96.5)
Appropriate referral	77/81	95.1 (87.8, 98.6)
Given nurse (educator) referral	81/81	100 (95.5, 100)
Given follow-up appointment	62/81	76.5 (65.8, 85.2)
Off-EML^b^	18/81	22.2 (13.7, 32.8)

All confidence intervals calculated using the Wilson score interval method.

^a^Appropriate diagnostic process: correctly doing all diagnostic practices such as a second confirmatory glucose test when necessary based on the first result. See *Outcomes* section for full details

^b^Off-EML = patients prescribed drugs not on the Essential Medicine List

We found no evidence that appropriate management varied by sex, age, UHC or type of NCD (Table 5). Nor did the individual components of appropriate management differ significantly between these groups, except that the diagnostic process was substantially poorer in UHC2 than UHC1 (Table 6). However, we lacked power to detect anything other than large effects. We also found that around one fifth of patients were prescribed drugs not on the Essential Medicine List (EML), but there were no significant differences by sex, age, UHC or NCD type.

**Table 5:** Adjusted odds ratios for associations between patient-level and facility-level variables and whether NCD patients were appropriately managed

Variable	AOR^a^ (95% CI); *P* value
Patient sex: female (vs male)	0.54 (0.19, 1.56); 0.26
Patient age	0.98 (0.93, 1.02); 0.29
Patient diagnosis: diabetes (vs HTN)	1.57 (0.41, 5.99); 0.51
Patient diagnosis: diabetes & HTN (vs HTN)	2.29 (0.37, 14.4); 0.38
UHC 2 (vs 1)	0.36 (0.09, 1.41); 0.14

^a^AOR = Adjusted odds ratios, obtained from a multiple logistic regression of NCD patient appropriate management in relation to patient sex (male or female), patient age (continuous), UHC facility (1 or 2) and patient diagnosis (diabetes, hypertension or both). The AOR for age, as a continuous variable, represents the relative (multiplicative) change in the odds of the outcome for every additional year older a patient is

**Table 6:** Adjusted odds ratios for associations between patient-level and facility-level variables and whether NCD patients received different appropriate management components

Variable	Appropriate management component				Additional management factor
	Appropriate diagnosis	Drug initiation	Appropriate referral	Given follow-up 22 pt.	Off-EML^b^
	AOR (95% CI); *P* value	AOR (95% CI); *P* value	AOR (95% CI); *P* value	AOR^a^ (95% CI); *P* value	AOR (95% CI); *P* value
Patient sex: female (vs male)	0.74 (0.23, 2.38); 0.61	0.54 (0.19, 1.56); 0.26	0.88 (0.10, 8.10); 0.91	0.45 (0.12, 1.69); 0.24	4.18 (0.84, 20.9); 0.08
Patient age	1.00 (0.95, 1.04); 0.88	0.98 (0.93, 1.02); 0.29	1.01 (0.92, 1.11); 0.87	0.97 (0.92, 1.02); 0.28	1.03 (0.98, 1.09); 0.24
UHC 2 (vs 1)	0.18 (0.06, 0.56); 0.003	0.36 (0.09, 1.41); 0.14	–	0.59 (0.13, 2.67); 0.50	3.8 (0.64, 22.43); 0.14
Diabetes (vs HTN)	–	1.57 (0.41, 5.99); 0.51	1.18 (0.09, 15.29); 0.90	3.68 (0.83, 16.3); 0.09	0.17 (0.03, 1.04); 0.06
Diabetes & HTN (vs HTN)	–	2.29 (0.37, 14.41); 0.38	0.42 (0.02, 7.97); 0.56	5.21 (0.45, 60.4); 0.19	4.85 (0.6, 38.95); 0.14

Note the management component *attending the NCD nurse (educator) for counselling* was not included in logistic regression analyses as all patients received this component

^a^AOR = adjusted odds ratios obtained from multiple logistic regressions for each appropriate management component outcome in relation to patient sex (male or female), patient age (continuous), UHC facility (1 or 2) and patient diagnosis (diabetes, hypertension or both). However, some AORs are missing where the relevant logistic regression model was not able to estimate all coefficients appropriately given the data. Where this occurred the model was refitted without the relevant problematic variable and the results presented are from these ‘reduced’ models. The AORs for age, as a continuous variable, represents the relative (multiplicative) change in the odds of an outcome for every additional year older a patient is

^b^Off-EML = patient prescribed drugs not on the Essential Medicine List

We also found that around one fifth of patients were prescribed drugs not on the EML.

Limited numbers of patients attended, particularly in UHC2. Patient concerns with having to take medicines indefinitely, particularly when they were not available routinely and for free, influenced their willingness to come to the UHC to be diagnosed, inhibiting early diagnosis:

‘The patient is afraid of taking medicines continuously. There are many who have a family history of diabetes; either the mother or the father had diabetes. Even after knowing it, they do not consult with a doctor because of their fear. Eventually, later, when the patient gets admitted to the hospital for other complications, they are then diagnosed with hypertension or diabetes during a routine check-up. The treatment starts at that time, which was supposed to be start earlier.’ OPD Doctor, UHC2

Low attendance was also due to limited awareness of the availability of the new NCD service, reflecting the challenge facing health services as they transition to provide these new services.

‘This awareness has not been developed among the people yet. For this reason, we are not getting all the patients completely. If we get all of them then the effectiveness of the NCD clinic would improve.’ NCD Doctor, UHC2

The unfamiliarity of the concept of long-term chronic care was evident, further exemplifying problems of transition of primary care services from acute to long-term care:

‘It is difficult to make them understand … they think, “If I take medicines for 7 days or 10 days, I will be cured”. So, it is difficult to make them understand that this [the treatment procedure] is a long process and it is impossible for us to cure them but we can control the disease.’ OPD Doctor, UHC2

However, the demand and need for a quality NCD service was evident, with attending patients showing a preference for NCD services delivered in the UHCs rather than in the private sector.

Patients were generally happy with the lifestyle advice received, although several would have preferred greater use of visual images within the patient leaflets, particularly of the food recommendations.

## Discussion

There were many problems implementing NCD care: shortages of staff, appropriate medication and diagnostic equipment; and challenges delivering interactive training with hands-on practice. These implementation deficiencies meant patients were unwilling to attend for diagnosis or follow-up and doctors were unwilling to provide follow-up appointments. Challenges with the provision of quality training [23] to the NCD doctors and nurses may partially explain problems with appropriate diagnosis and management. A common issue was that a second BP measurement was not taken (or not recorded) prior to diagnosing hypertension. One high BP may be due to anxiety, and if not confirmed in a repeat measurement may lead to over-diagnosis of hypertension. In addition, patients diagnosed with diabetes rarely had their BP checked (or recorded). These quality of care issues will need to be addressed through revision of training exercises and supervision/ monitoring.

The challenges of encouraging patient lifestyle behaviour change using nurses within NCD corners were evident. Ensuring proper training was a particular issue. This may be due to lifestyle change being given low priority and the hierarchical nature of the Bangladesh health system [24]. Given the scale of the NCD epidemic in Bangladesh and other South Asian countries, support to patients to change their lifestyles for secondary prevention is key [25]. Encouraging patients’ active involvement (‘self-management’) is particularly important given the constraints the health system faces appropriately managing patients within primary care [3].

Despite these limitations, the NCD clinics appropriately managed approximately half of patients with diabetes and/or hypertension, failure being largely due to an incomplete diagnostic process (e.g. missing hypertension in a diabetic), or not giving a follow-up appointment. While there is still clearly great room for improvement, this study shows a relatively simple intervention can begin to provide appropriate care to a large proportion of patients, for whom previously there was no suitable care available within this setting. A key principle in our study was to embed the intervention into government practice to facilitate scale-up. This approach has led to dissemination of our materials along with training to doctors at 50 NCD clinics across Bangladesh.

While our study was not powered to look at gender differences, evidence from other studies indicates that women are less likely than men to be appropriately diagnosed for CVD [26]. Further exploration of this issue in future studies is recommended.

Limited number of facilities and inadequate sample size are major limitations of the study. Care should be taken extrapolating the findings of this study, due to the small number of sites, and their potential lack of representativeness. Nonetheless, the qualitative findings provide a clear indication of practitioners’ experiences in attempting to establish a comprehensive NCD clinic. While NCDs have gained increased focus within Ministries of Health, operationalising NCD policies so that drugs, equipment and training are available in primary care clearly remains a challenge. Once facilities are fully functional in their delivery of NCD services, future research should focus on evaluating the effectiveness of the intervention across a representative range of sites.

To provide good NCD care, a trained doctor must be permanently stationed at the NCD clinic, together with a nurse to provide health education, and a regular supply of essential NCD drugs, diagnostic and recording equipment. In addition, to meet the challenges of implementing complex NCD care to ensure appropriate management, training must contain a substantial practical element. Advice on lifestyle behaviour change is vital if patients are to be able to manage their condition and avoid complications. Appropriate and available provision of lifestyle behaviour change counselling requires due recognition of the roles of nursing staff, and training to enable them to deliver this part of the intervention.

## Conclusion

A clinical guide, skill-based training and recording package can be implemented in routine primary care and lead to effective management of at least half of diabetic and hypertensive patients. However, considerable health systems challenges must be addressed before the majority of patients can be managed appropriately; in particular, ensuring regular drug supplies, availability of dedicated doctors and health educationalists, and effective hands-on practical training to ensure staff have the skills they need to address these complex long-term conditions.

## Abbreviations

BADAS: Diabetic Association of Bangladesh
BIRDEM: Bangladesh Institute of Research and Rehabilitation of Diabetes, Endocrine and Metabolic Disorders
BP: Blood pressure
CI: Confidence Interval
COMDIS-HSD: Communicable Disease-Health Service Delivery
COPD: Chronic obstructive pulmonary disease
CVD: Cardiovascular Disease
EC: Exit client
EML: Essential Medicine List
EOC: Emergency obstetric care
ESP: Essential service package
FBG: Fasting blood glucose
HTN: Hypertension
ID: Identity
IDF: International Diabetes Federation
IDI: In-depth interview
LMIC: Low- and middle-income country
NCD: Non-communicable disease
OPD: Outpatient department
RBG: Random blood glucose
UH&FPO: Upazila Health & Family Planning Officer
UHC: Upazila Health Complex
UK: United Kingdom
WHO: World Health Organisation
